# Lactoferrin in Aseptic and Septic Inflammation

**DOI:** 10.3390/molecules24071323

**Published:** 2019-04-03

**Authors:** Maria Stefania Lepanto, Luigi Rosa, Rosalba Paesano, Piera Valenti, Antimo Cutone

**Affiliations:** 1 Department of Public Health and Infectious Diseases, University of Rome La Sapienza, 00185 Rome, Italy; mariastefania.lepanto@uniroma1.it (M.S.L.); luigi.rosa@uniroma1.it (L.R.); piera.valenti@uniroma1.it (P.V.); 2 Microbo S.R.L., 00153 Rome, Italy; info@microbosrl.it; 3 Department of Biosciences and Territory, University of Molise, 86090 Pesche, Italy

**Keywords:** lactoferrin, inflammation, iron homeostasis, anemia of inflammation, preterm delivery, alzheimer’s disease, type 2 diabetes, *Chlamydia trachomatis* infection, cystic fibrosis, inflammatory bowel disease

## Abstract

Lactoferrin (Lf), a cationic glycoprotein able to chelate two ferric irons per molecule, is synthesized by exocrine glands and neutrophils. Since the first anti-microbial function attributed to Lf, several activities have been discovered, including the relevant anti-inflammatory one, especially associated to the down-regulation of pro-inflammatory cytokines, as IL-6. As high levels of IL-6 are involved in iron homeostasis disorders, Lf is emerging as a potent regulator of iron and inflammatory homeostasis. Here, the role of Lf against aseptic and septic inflammation has been reviewed. In particular, in the context of aseptic inflammation, as anemia of inflammation, preterm delivery, Alzheimer’s disease and type 2 diabetes, Lf administration reduces local and/or systemic inflammation. Moreover, Lf oral administration, by decreasing serum IL-6, reverts iron homeostasis disorders. Regarding septic inflammation occurring in *Chlamydia trachomatis* infection, cystic fibrosis and inflammatory bowel disease, Lf, besides the anti-inflammatory activity, exerts a significant activity against bacterial adhesion, invasion and colonization. Lastly, a critical analysis of literature in vitro data reporting contradictory results on the Lf role in inflammatory processes, ranging from pro- to anti-inflammatory activity, highlighted that they depend on cell models, cell metabolic status, stimulatory or infecting agents as well as on Lf iron saturation degree, integrity and purity.

## 1. Iron and Inflammation

In all living cells, iron is necessary for pivotal metabolic processes such as energy production, DNA synthesis/repair/transcription, oxygen transport/storage and drug detoxification. These biological processes rely on iron’s ability to accept/donate electrons by redox-fluctuation between divalent ferrous (Fe^2+^) and trivalent ferric (Fe^3+^) ions, which in intracellular habitat constitute the iron pool (IP). Of note, Fe^3+^ is less soluble than Fe^2+^ and the precise cytosolic quantization of Fe^3+^ with respect to Fe^2+^ (Fe^3+^/Fe^2+^ ratio) is still unknown [1]. However, free available not bound-Fe^3+^ is very reactive and potentially toxic due to the induction of reactive oxygen species (ROS) formation via the Fenton and Haber-Weiss reactions [2]. ROS formation, causative of damages to lipids, nucleic acids and proteins, is involved in the cell, tissue and organ oxidative stress and injury with consequent activation of either acute or chronic inflammatory processes implicated in multiple degenerative clinical conditions including the development of cancer, obesity, diabetes and aging. Inflammation is a defensive mechanism addressed to eradicate unsafe stimuli, thus contributing to the repair of damaged cells or tissues. In particular, acute inflammation occurs promptly after the injury and it is characterized by local vasodilatation and increased vessel permeability to ameliorate molecules and cells delivery to help damaged parenchyma repair. However, acute inflammation can converge into the chronic state if the injury persists or the individual’s immune system is impaired. Differently, chronic inflammation involves the cycling of destructing and healing processes in tissues suffering from persistent and recurring damage.

To counteract iron chemical reactivity, all organisms have evolved highly specialized systems to bind and transport the metal avoiding the formation of either ROS or insoluble precipitates due to free available iron [3]. Moreover, several enzymes belonging to oxidases and reductases family control iron oxidative state [4].

Since there is no specific excretory system for iron in mammals, the correct balance of this metal between tissues/secretions and blood, defined iron homeostasis, is mainly regulated by two different pathways: dietary iron absorption from the enterocytes (1–2 mg) and iron recycling from the lysis of senescent erythrocytes by macrophages (about 20 mg/day) [5,6]. Enterocyte iron absorption is primarily carried out through an apical reductase, the duodenal cytochrome b (Dcytb). The resulting ferrous iron is internalized by apical divalent metal transporter 1 (DMT1). In the cytoplasm, ferrous ions belonging to IP are sequestered and oxidized by ferritin (Ftn). According to the body’s request, iron is newly reduced inside Ftn and ferrous ions are exported. On the baso-lateral side of enterocytes, ferroportin (Fpn), the sole protein able to export iron from cells to blood, found also in macrophages, hepatocytes and placental cells [7], releases Fe^2+^ to hephaestin (Heph), which, in turn, oxidizes iron to allow Fe^3+^ binding to transferrin (Tf) in the blood. Tf is a glycoprotein able to bind two ferric ions per molecule. Iron transport from blood to cells occurs through the binding between Fe-Tf and its receptor 1 (TfR1), leading to endosome formation. In the acidified endosome iron is released from Tf and translocated, after the reduction by six-transmembrane epithelial antigen of the prostate 3 (STEAP3), via DMT1 into the cytoplasm where it can be utilized for cell metabolism [8] or sequestered by Ftn. In circulating macrophages, the iron import also occurs through the binding between Fe-Tf and TfR1. Inside the macrophages, iron, deriving from both Tf-mediated import and lysis of senescent erythrocytes, is sequestered by Ftn and then, according to the request, exported by Fpn acting in partnership with ceruloplasmin (Cp), another ferroxidase similar to Heph [4]. Of note, ferroxidase activity by Cp has been demonstrated to be crucial for the stabilization of Fpn on the plasma membrane [4]. In fact, some pathological conditions, characterized by the absence of Cp activity (i.e., aceruloplasminemia), show a prominent induction of Fpn internalization and degradation, with consequent accumulation of intracellular iron and promotion of ROS production [4,9,10].

The main regulator of iron homeostasis is hepcidin, a 25 amino acid cationic peptide hormone, found in plasma [11] and urine [12,13]. Hepcidin, modulated by iron stores as well as by hypoxia, is involved in the internalization and degradation of Fpn [14]. Consequently, high hepcidin levels, hindering iron export by Fpn, lead to intracellular iron overload as well as to a decrease of the iron import systems as Dcytb, DMT1 and TfR1 [15]. Conversely, hepcidin low levels, unable to trigger Fpn degradation, restore iron export and import [6,16].

In inflammatory status where high levels of pro-inflammatory cytokines, as interleukin (IL)-1α, IL-1β and IL-6, are detected, iron export is significantly impaired by hepcidin up-expression [17,18] and Fpn down-regulation [19,20]. However, it cannot be excluded that the down-regulation of Fpn might be mediated by IL-6 also in a hepcidin-independent way [21,22] as demonstrated in beta-thalassemic pregnant and non-pregnant women [23].

Overall, iron homeostasis disorders are so closely connected to inflammatory disorders, that it is difficult to distinguish which is the cause and which is the effect. Moreover, inflammation can take place in the presence of the infection, where high levels of free available iron favor bacterial growth and severity of infection. As matter of fact, iron is also essential for the life and multiplication of pathogenic microorganisms except for some commensal bacteria as *Lactobacillus* is able to grow without this metal.

In addition, inflammation arises also in the absence of infection as a natural response following tissue’s damage or excessive cell death [24]. The tissue injuries unrelated or related to infections generate a strong immune response resulting in the release of pro-inflammatory bioactive compounds leading to the consequent development of systemic diseases such as systemic inflammatory response syndrome or sepsis. The cytokines and chemokines are the primary immune-mediators depending on the recruitment of neutrophils, macrophages and dendritic cells, while secondary mediators, as complement system, are activated consequently. Several connections among immune cells, primary and secondary mediators defend host against systemic infections or pathological destructive inflammation thus supporting damaged tissue repair [25]. In this review, we’ll refer to infection-unrelated inflammation as “aseptic inflammation” and to infection-related inflammation as “septic inflammation”.

## 2. Lactoferrin

As already reported, highly specialized systems have been evolved by all organisms to bind iron and, in mammals, lactoferrin (Lf) plays a key role. Lf is a 80 kDa glycoprotein able to bind two ferric ions per molecule, identified in bovine milk in 1939 and isolated in 1960 from both human [26,27] and bovine milk [28].

The Lf primary structure has been characterized in multiple species [29]. Human Lf (hLf) is a single polypeptide chain constituted of 691 amino acids [30], while bovine Lf (bLf) is constituted of 689 amino acids [31]. HLf and bLf as well as recombinant hLf (rhLf) are organized in two highly homologous structured lobes, the N-lobe and the C-lobe. Each lobe is capable of binding reversibly a single ferric ion with high affinity (Kd ~ 10^−20^ M). The ferric ligands are highly conserved in iron-binding proteins [32,33] and, in Lfs, they are identical in both lobes: one aspartic acid, two tyrosines and one histidine.

HLf, bLf and rhLf can assume two principal conformational states: the open iron-free (apo-Lf) and the closed iron-bound (holo-Lf) [30,34]. HLf and bLf iron-saturated closed forms show a higher thermal stability and a greater resistance to proteases’ digestion compared to the unsaturated open ones [35,36] and retain ferric iron at pH values (3.0) lower than Tf, a peculiar iron-shuttle glycoprotein [37].

Typically, about 15–20% of iron saturation rate characterizes the Lf native form in physiological conditions, while in pathological ones holo-form is frequently present. Studies have shown that apo- and holo-Lf forms can exert dissimilar functions [3,36,38,39]. Therefore, it is important to take into account Lf iron saturation rate as well as integrity and purity when applied in in vitro and in vivo models [36]. Of note, Lfs iron binding ability strongly contributes to innate immune defence against uncontrolled ROS production. As recently reviewed by Kruzel et al. [25], Lf is able to control the physiological balance of ROS production and the rate of their elimination by directly regulating key antioxidant enzymes, thus exerting its potent anti-inflammatory activity against ROS-mediated cell and tissue injuries.

Moreover, Lf is a cationic charged protein (pI around 9) with a high tendency to interact with anionic charged molecules, either in solution or at the cell surface. Lf positive charge is predominantly due to the highly abundance of basic amino acids in the N-lobe, and particularly in two peptide sequences, lactoferricin (residues 1–47 in hLf and residues 17–41 in bLf) [40] and lactoferrampin (residues 269–285 in hLf and residues 268–284 in bLf) [41], which have been described as the major interactors to Lf molecular targets.

Lastly, all mammalian Lfs identified so far are glycosylated, with the N-glycan profiles varying among species [42]. HLf and bLf possess three (asparagine (Asn) 138, 479 and 624) and five (Asn233, 281, 368, 476, and 545) potential *N*-glycosylation sites, respectively [43]. Among these, only two sites undergo glycosylation in hLf: Asn138 and Asn479 [44] and four sites in bLf: Asn233, Asn368, Asn476, and Asn545 [31]. *N*-linked glycosylation has been described to protect both hLf and bLf from tryptic proteolysis [45]. Moreover, Lfs glycans have been described to be involved in different cellular pathways, including cell adhesion and receptor activation [46] as well as in protecting host from pathogen and viral attacks [42].

In humans, hLf is constitutively synthetized and secreted by glandular epithelial cells, at different concentrations [47,48,49]. Moreover, hLf concentration is strongly influenced by inflammatory processes that recruit neutrophils which, in turn, secrete secondary granules containing Lf. Of note, 10^6^ neutrophils release 15 µg of hLf.

HLf shows a high sequence similarity with bLf [31] which possesses identical multifunctions, as anti-bacterial, anti-biofilm, anti-fungal, anti-viral and anti-parasitic, anti-oxidative stress, immunomodulatory and anti-inflammatory activities strictly related to intracellular iron overload [3,50].

Therefore, the majority of the in vitro and in vivo studies have been performed by using commercial bLf (cbLf), generally recognized as a safe substance (GRAS) by the Food and Drug Administration (FDA, USA) and available in large quantities. Concerning metal binding activity, hLf and cbLf, even if at lower affinity than Fe(III), are able to chelate other metal ions such as Al(III), Ca(II), Cu(II), Mg(II), Mn(II), Zn(II) [29]. Ca(II) can be also bound by the carboxylate groups of sialic acid present on bLf glycan chains. This process has been described to be involved in the release of Gram-negative lipopolysaccharide (LPS), thus contributing to the bLf bactericidal activity [51].

Hence, the efficacy of hLf, rhLf, bLf and cbLf in exerting their multiple functions can be critically influenced by purity, integrity, iron or other metal ions saturation rate, *N*-glycosylation and sialylation profiles and finally by their ability to enter inside the nucleus [36].

Another pivotal parameter influencing the Lfs efficacy is the in vivo bioavailability. In this respect, the oral administration of bLf should be performed before meals to avoid the protein degradation due to the low pH of gastric juice during digestion (about 1.5). Conversely, at pH about 4, characteristic of gastric juice before meals, 90% of the administered bLf arrives undigested to the intestine [23].

Other than anti-microbial activity, reviewed elsewhere [3,50], both hLf and bLf perform other pivotal biological activities, ranging from modulation of cell cycle, migration and differentiation to anti-cancer and immunomodulatory ones [52]. These biological functions, exerted by Lfs, are strictly dependent from target cell as well as from Lf interaction with different host molecules, such as glycosaminoglycans (GAGs) [53], heparan sulfate proteoglycans [54] and DNA [55]. Concerning hLf receptors (hLfRs), they have been characterized in different cell types and include Intelectin-1 (ITLN1), LDL receptor-related protein (LRP), asialoglycoprotein receptor (ASGPR), CD14 and nucleolin [56,57]. All hLfRs have been described to be differentially expressed according to tissue and cell type [57]. ITLN1 is a high affinity Lf receptor, firstly discovered in the intestinal epithelium, involved in hLfs uptake from the digestive tract [58]. ITLN1 has been found on the intestinal brush border, Paneth and goblet cells [59] as well as in biliary epithelium [60]. Differently, a low specificity receptor for hLf, LDL receptor-related protein 1 (LRP1), can bind multiple targets and it is principally expressed in hepatocytes, neurons, smooth muscle cells, fibroblasts and cholangiocytes [60]. Of note, hLf, after binding with the relative receptor, is internalized through clathrin-mediated endocytosis and translocates into the nucleus [61] thus modulating gene expression in enterocytes [62] or triggering intracellular signaling pathways depending on its iron-saturation rate [39]. Concerning other LfRs, CD14 was exclusively found on monocytes, ASGPR in liver and nucleolin in lymphocytes [56]. Thus, due to the receptor specificity, hLfs can exert multiple and differential functions depending on the cell system it acts upon.

Furthermore, the discovery of hLf ability to enter into the host nucleus [58,61] and to bind to DNA open up new perspectives on the role of this glycoprotein in the direct modulation of gene expression. In particular, Suzuki et al. [58] have demonstrated the importance of the sole N-lobe, compared to C-lobe, for hLf binding, internalization and targeting to the nucleus. Therefore, endocyted Lf can be targeted to the nucleus where it could act as a gene transcriptional regulator or trans-regulator [63]. In this respect, both ITLN1 and surface nucleolin were shown to be possible vectors for this targeting [64,65].

Importantly, it has been demonstrated that orally administered Lf can enter systemic circulation through intestine via lymphatic pathway. In particular, transepithelial transport of Lf into systemic circulation has been documented in animal models [66,67]. Therefore, Lf oral administration plays pivotal physiological functions not only in the intestine but also at systemic levels, including its important anti-inflammatory activity.

Of note, the interaction between bLf and hLfRs has been disclaimed until 2008, when Shin et al., [68] showed that bLf was able to bind to hLfRs. Later on, similarly to that observed with hLf [58,61], bLf was described to be able to enter inside the nucleus of host human cells [5], thus finally elucidating the possible molecular mechanism through which it could act as a potent anti-inflammatory agent. All these observations have highlighted the great potential of bLf to act as a bioequivalent of hLf in many application fields.

Overall, whereas potential molecular mechanisms are still under investigation, Lfs interaction with cell receptors as well as its targeting to the nucleus seem to be the most reasonable mechanisms through which Lfs can exert its pleiotropic functions, including immunomodulatory and anti-inflammatory ones.

## 3. Lactoferrin Against Aseptic Inflammation

Inflammation is a protective and coordinated approach acting to restore tissue integrity. In particular, aseptic inflammation, characterized by redness, heat, swelling, and pain of the tissues, can be triggered by physical, chemical, metabolic or genetic dangerous stimuli.

Even if the relative pathways are not completely clear, aseptic inflammation is caused by the rupture of cell plasma membrane liberating intracellular substances. Consequently, necrotic cell death can generate aseptic inflammation while apoptosis is a cell death program well organized and immunologically silent [69]. In fact, the apoptotic process does not lead to the immediate cell membrane rupture and release of intracellular substances (signals) inducing inflammation, as described for necrosis. Instead, before membrane disruption occurs, apoptotic cells are rapidly phagocytized by local macrophages. In addition, apoptosis can direct macrophages to the production of several anti-inflammatory cytokines such as IL-10 and transforming growth factor (TGF)-β [70]. It has been proposed that the different response to necrotic or apoptotic cell death could be due to an ancestor host adaptation to potentially dangerous (for necrosis) or physiologic remodeling (for apoptosis) processes [71]. According to this hypothesis, a fast inflammatory activation in necrotic sites can be potentially beneficial whereas not for the apoptotic ones. Overall, in persistent aseptic inflammatory conditions, the ratio between positive and negative effects becomes strictly critical, with the host facing more damages than benefits. Definitely, for the host, inflammation is a double-edged sword.

In these conditions, the best way to block the detrimental persistence leading to chronic inflammatory processes would be achieved with an anti-inflammatory therapeutic approach. In this respect, it is important to consider the anti-inflammatory activity of Lf [50] as well as its influence on the apoptotic process [72,73].

### 3.1. Lactoferrin Against Anemia of Inflammation

Anemia of inflammation (AI, also called anemia of chronic disease) is typically characterized by low hematological parameters despite adequate iron stores, as evidenced by normal-to-elevated levels of serum Ftn (sFtn), associated to high levels of pro-inflammatory cytokines [74].

AI is common in patients suffering from chronic diseases with prolonged inflammation due to aseptic diseases as cancer and obesity [75,76].

The link between inflammation and anemia rely on the pro-inflammatory cytokines-mediated down-regulation of the iron exporter Fpn. The Fpn down-regulation by IL-6, either hepcidin-depend or independent [23], is the main step responsible for cell-to-blood iron export inhibition, leading to iron overload in enterocytes and macrophages and iron deficiency in blood. Subsequently, AI is established [77]. Furthermore, the persistence of inflammatory stimuli exacerbates the anemic status by inhibiting erythroid cell differentiation, thus worsening AI [77].

Originally, AI has been considered as a host protective mechanism to hinder free iron availability to bacteria in the blood [2,78]. However, in light of bacteria ability to invade and survive inside host cells, including macrophages, this conception should be critically revalued. Indeed, intracellular iron overload could be involved both in intracellular pathogens’ growth and in increasing the severity of infection [20]. As referred, iron and inflammation are closely connected as iron disorders increase inflammation and vice versa. Hence, it is crucial to counteract the persistence of the inflammatory status thus rebalancing physiological iron levels between tissues/secretions and blood.

Since AI is a secondary manifestation of inflammatory disorders, anti-inflammatory therapeutic approaches should be mainly addressed to restore physiological iron homeostasis. So far, other than classical iron supplementation, almost all therapeutic strategies, principally based on erythropoiesis-stimulating agents and erythrocyte transfusions, are not fully efficient against AI [79,80,81]. Among the putative innovative therapies, it has been proposed the use of Fpn agonists or hepcidin antagonists [16,74]. Recently, bLf has been described to be able to either counteract inflammatory disorders by down-regulating IL-6 and contemporary, by up-regulating Fpn expression, to redistribute endogenous iron between tissue/secretions and blood [19,20,23,82]. The therapeutic use of bLf against AI has been firstly described by our group [23,82].

Since 2006, our clinical study on the efficacy of 30 days of bLf oral administration compared with classical ferrous sulfate therapy in anemic pregnant women has represented a mile stone in the bLf experimentation against anemia [83]. In particular, pregnant women treated with 100 mg of 20–30% iron-saturated bLf two times a day before meals (corresponding to 70–84 µg/day of elemental iron) showed a significant rescue of the concentration of both hemoglobin (Hb) and total serum iron (TSI) compared to pregnant women treated with 329.7 mg of ferrous sulfate once a day during meals (corresponding to 105 mg of elemental iron). The discrepancy between elemental iron supplementations and the efficacy in increasing hematological parameters by bLf vs. ferrous sulfate managements has led us to speculate that the efficacy of bLf in curing AI was not directly linked to the iron supplementation itself but to a more intricate mechanism involving this glycoprotein in iron and inflammatory homeostasis.

In our subsequent clinical trials, we have treated pregnant and non-pregnant women suffering from hereditary thrombophilia (HT) and affected by AI [23,82]. HT is an inherited genetic condition, involving aberrant coagulation processes, predisposing the subjects to venous thrombus formation [84,85]. In pregnancy, this status is exacerbated by the increased activity of several coagulation factors, such as VII, VIII, X and the von Willebrand factor, as well as higher levels of fibrinogen [86] and thrombin generation markers, such as prothrombin F1 and F2 [87]. Furthermore, several components of the inflammatory response, including IL-6, IL-8 and tumour necrosis factor (TNF)-α, result activated [88]. These disorders can converge in increased maternal and fetal complications, such as placental abruption, miscarriage, pre-eclampsia, fetal growth limitation and intrauterine death and stillbirth [87]. Therefore, in order to prevent and limit venous thromboembolism risk as well as hypercoagulability-associated miscarriage, HT pregnant women undergo low molecular-weight heparin and low dose aspirin treatments.

In this respect, bLf treatment was found to be significantly efficient in curing AI in HT pregnant and non-pregnant women compared to the classical ferrous iron therapy [23,82]. In particular, after 30 days of bLf treatment, both HT pregnant and non-pregnant women showed a significant recovery in the total number of red blood cells (RBCs) as well as in the concentration of serum Hb, TSI and Ftn. Notably, bLf management was also efficient in significantly reducing serum IL-6 and hepcidin levels with respect to the higher basal ones [23]. Importantly, in our opinion, the bLf-mediated hepcidin down-regulation can be linked to a compensatory mechanism exerted by this glycoprotein addressed to restore the physiological cell-to-blood iron export by up-regulating Fpn expression. Differently from bLf, ferrous sulfate treatment showed no significant increase of RBCs and Hb even if elemental iron provision was a thousand-fold higher than the one supplied by bLf (about 100 mg/day versus about 80 µg/day, respectively). Moreover, ferrous sulfate treatment failed in down-regulating both serum IL-6 and hepcidin concentrations [23]. This failure can be easily explained by the fact that ferrous sulfate management has never been demonstrated to be able to rebalance hepcidin or Fpn-expression, neither directly nor indirectly by down-regulating IL-6 levels, during inflammatory status.

Hence, even if more studies are needed to unveil the molecular mechanisms underlying bLf anti-inflammatory activity, this evidence strongly supports our idea that the bLf efficacy in curing AI is directly connected to its ability to decrease IL-6 synthesis [4,50] thus modulating hepcidin and/or Fpn, the most pivotal actors in systemic iron homeostasis [16,74].

**Figure 1:**
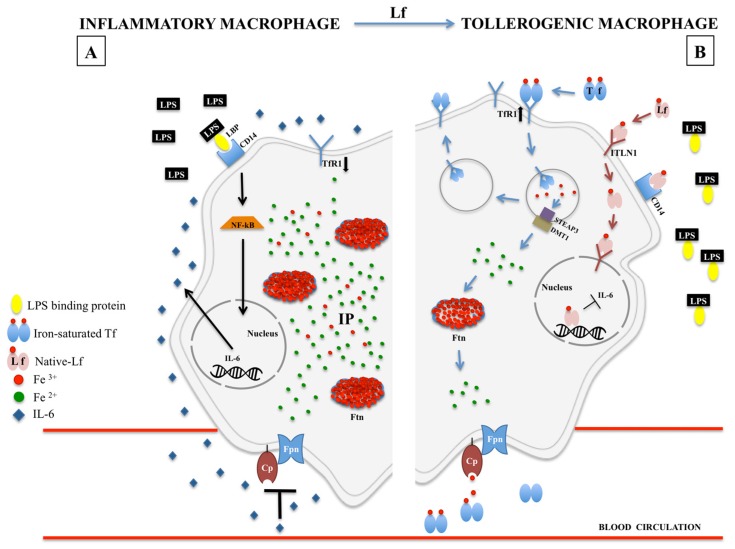
Iron homeostasis in LPS-inflamed macrophages, in the absence (**A**) or presence (**B**) of lactoferrin. (**A**) LPS stimulation, triggered by LPS-binding protein (LBP) and CD-14, induces the nuclear translocation of cytoplasmic NF-κB which in turn induces IL-6 synthesis. Serum IL-6 down-regulates Fpn/Cp axis, inhibiting iron export from the macrophage to blood circulation. Consequently, the increase of intracellular iron pool (IP) triggers the up-regulation of intracellular Ftn and the down-regulation of TfR1 expression thus hindering additional intracellular iron accumulation. (**B**) Lf-mediated inflammatory-to-tolerogenic macrophagic shift. Lf is internalized by ITLN1-mediated endocytosis and translocated into the nucleus where it inhibits IL-6 synthesis. As a cascade, the inhibition of IL-6 synthesis restores Fpn/Cp-mediated iron export from the macrophage to blood circulation. Consequently, the decrease of intracellular IP triggers either the inhibition of Ftn synthesis and the up-regulation of TfR1, thus restoring the physiological iron balance between reticuloendothelial system and blood. LPS: lipopolysaccharide; LBP: LPS binding protein; NF-κB: nuclear factor-κB; IL-6: interleukin-6; IP: iron pool; Fpn: ferroportin; Cp: ceruloplasmin; Ftn: ferritin; Tf: transferrin; TfR1: transferrin receptor 1; Lf: lactoferrin; ITNL1: intelectin-1; STEAP3: six-transmembrane epithelial antigen of the prostate 3; DMT1: divalent metal transporter 1.

Recently, our group has demonstrated in in vitro experiments on human inflamed macrophages that bLf treatment determines a macrophagic shift from M1 to M2 phenotype. As matter of fact, following inflammatory stimuli, macrophages polarize into M1 phenotype, characterized by the synthesis of pro-inflammatory cytokines, including IL-6, as well as by the dysregulation of the main iron-related proteins, as the down-regulation of Fpn/Cp axis, TfR1 and the up-regulation of cytosolic Ftn [20,89,90]. All these changes lead to the blockade of iron recycling to blood by macrophages, the major iron source for the body deriving from the lysis of senescent erythrocytes [20]. We have proved that bLf reverts iron disorders induced by inflammatory stimuli by down-regulating IL-6 synthesis and up-regulating Fpn expression, thus restoring the physiological cell-to-blood iron export [19]. Importantly, bLf, other than Fpn expression, is able to modulate all the iron-related proteins, up-regulating TfR1, Cp and down-regulating cytosolic Ftn [20] (Figure 1).

Our current finding on the linkage between bLf anti-inflammatory activity and its capacity in counteracting the pathological intracellular iron overload by restoring Fpn-mediated iron efflux from tissue/secretions to blood can be considered a realistic molecular mechanism explaining the bLf efficacy in curing AI [23,82].

### 3.2. Lactoferrin Against Inflammation-Related Preterm Delivery

Many studies have explored the relationship between inflammation and pregnancy [91,92]. Pregnancy adverse outcomes, such as miscarriage, abortion and preterm delivery (PTD), are strictly associated with inflammatory processes and, in particular, to cytokine imbalance, and in this respect, IL-6 seems to play a key role as activator of acute phase immune response [93]. Indeed, in physiological pregnancies, the balance between T helper cells 1 (Th1) and Th2 is strongly shifted toward Th2, playing a defensive role in the fetal-maternal relationship [94]. This balance is perturbed by inflammatory processes causing a shift toward Th1 predominance, characterized by pro-inflammatory cytokines synthesis involved in the stimulation of cervical prostaglandin secretion, uterine contractility, PTD [5], intrauterine growth restriction, low birth-weight and inferior neonatal health [95] as well as in the establishing of iron homeostasis disorders as anemia, often occurring during pregnancy [5,50,96]. Furthermore, high IL-6 levels in amniotic fluid (AF) have been associated with subsequent pregnancy loss [97] as well as to PTD and fetal growth restriction [98,99] thus suggesting that the primary efforts to prevent and counteract all these adverse outcomes should be addressed to counteract the underneath inflammatory disorders.

Several interventions, such as cervical cerclage and bed rest, as well as common therapies, such as beta mimetics, atosiban and calcium channel blockers were mostly ineffective in preventing PTD [100,101]. Conversely, bLf, due to its anti-inflammatory activity especially against IL-6 axis [50], has been demonstrated to be a promising candidate for PTD treatment.

Few studies have employed bLf to prevent PTD [5,102], a peculiar adverse outcome occurring before the 37th week of gestation, related to high rates of mortality and morbidity, comprising necrotizing enterocolitis, intraventricular hemorrhage, neurological deficit and respiratory distress syndrome [103]. As already remarked, aseptic or septic inflammatory processes represent risk factors for PTD [95,104,105,106]. In our clinical trial [5], we chose to treat PTD unrelated to cervico-vaginal infection in order to study only the actual anti-inflammatory activity of bLf distinguished from its anti-microbial function. The combined administration of oral and intravaginal bLf has been found to be an excellent treatment in preventing, without any side effects, PTD by decreasing both cervico-vaginal IL-6 and prostaglandin F2a (PGF2a), the main activators of both uterine contractions and membrane ruptures [5,107]. Importantly, in physiological conditions, the tight interplay between IL-6 and PGF2a is pivotal to regulate term delivery [108]. Indeed, the use of prostaglandin synthase inhibitors inhibits uterine contractility, thus extending the pregnancy length [109]. For this reason, the bLf intravaginal treatment, consisting of 100 mg of bLf every 8 h, was carried out until the 37th week of gestation thus avoiding any interference with physiological processes occurring in spontaneous term delivery. As matter of fact, within 2–7 days from the ending of bLf intravaginal therapy, all women gave birth thus indicating that the absence of bLf favoured the increase of IL-6 and PGF2a levels, predisposing the labor and delivery. Overall, bLf intravaginal administration was found to be an effective treatment against PTD by counteracting underneath inflammatory processes thus preventing the further shortening of cervical length and extending the pregnancy length [5]. Almost the same results were obtained by Locci et al. [102] in a longitudinal study where intravaginal bLf treatment, consisting of 300 mg bLf for 21 days in asymptomatic women at low risk for PTD, was effective in lowering cervico-vaginal IL-6 levels and improving cervical length [102]. Moreover, in other studies, conducted on pregnant women showing high levels of pro-inflammatory mediators in AF, a single intravaginal bLf administration (300 mg), 4 h or 12 h before amniocentesis, was surprisingly efficient in partially decreasing some mediators of pro-inflammatory processes, including IL-6 [110,111,112].

Overall, these data strengthen our claim for a possible protective role of this glycoprotein against inflammation-related to PTD.

### 3.3. Lactoferrin Against Inflammation in Alzheimer’s Disease

Alzheimer’s disease (AD) is an irreversible and progressive neurodegenerative disorder which principally affects the older adults. AD is characterized by early loss of memory, language disorders, loss of directionality and states of anxiety [113]. These conditions worsen in the late-stage of pathology leading to abnormal mental activities and a gradual loss of body functions [114]. At the molecular level, the brains of AD patients show a high deposition of extracellular β-amyloid protein (Aβ) forming senile plaques (SPs) and a hyperphosphorylation of tau proteins associated with microtubules in neurons causing the formation of neurofibrillary tangles (NFTs) [115]. In addition to SPs and NFTs, AD brain shows chronic inflammatory processes mainly around the amyloid plaques of activated microglia [116]. These microglia promote the synthesis of different pro-inflammatory cytokines, such as IL-1β, IL-6, TNF-α and interferon (IFN)-γ, which in turn induce the further formation of Aβ oligomers [117,118]. Moreover, either the increase of serum IL-6 and the decrease of the anti-inflammatory IL-10 levels were observed in AD patients [119]. The inflammation is one of the pathogenetic mechanisms in different neurodegenerative diseases and the detection of serum inflammatory mediators can help to diagnose AD at the preclinical stage and to monitor the progress of the disease [120].

Besides to neurological and serum inflammation, magnetic resonance imaging highlights an increase of iron content in the brains of AD patients [121]. Differently from iron deficiency which can cause irreversible developmental delays, the increase of free iron concentration, indicating a dysregulation of iron homeostasis, compromises brain development as well as its functions due to the increase of oxidative stress associated to higher ROS and nitric oxide synthase (NOS) production [117,118,122]. In the brain, iron regulates several functions as the maintenance of the high aerobic metabolic capacity of neurons, the synthesis of myelin, synthesis and metabolism of neurotransmitters and development of the neuronal dendritic tree [123]. Since iron overload plays a crucial role in the development and progression of AD, in recent years, the use of iron chelators has received much attention. However, the entry of almost all the drugs in the brain is complicated by the presence of the blood-brain barrier (BBB). Therefore, an ideal metal chelator to treat AD should cross easily the BBB. For this purpose, nanotechnological approaches have being studying [124] as well as new drug delivery methods such as intranasal administration [125].

Among iron chelators, deferoxamine (DFO) has given positive results in in vitro and in vivo studies. Intramuscular injections or oral or intranasal administration of DFO relieved the symptoms of AD patients compared with the placebo administration [126,127,128]. However, DFO presents some side effects, as neurotoxicity in the long-term treatments, and disadvantages, as poor bioavailability and gastrointestinal malabsorption [122,127,128,129,130].

Another important iron binding compound, hLf, is greatly up-regulated during neurodegenerative disorders, such as AD, and in elderly brains [131,132,133,134,135]. HLf seems to be expressed by neurons, astrocytes, microglia, and oligodendrocytes of the human brain [133,136]. The increase of hLf in the AD brain, due to the activated microglia, seems to be also related to the BBB receptor-mediated translocation of circulatory hLf [137], synthesized and released by neutrophils’ degranulation. However, this compensatory synthesis of hLf seems to be unable to counteract the pathogenesis of AD, mainly associated to oxidative stress and inflammatory process. As a matter of fact, recent studies have demonstrated that a further administration of Lf is active against AD.

Interestingly, Guo et al. [138] have demonstrated that intranasal rhLf administration decreases Aβ deposition and attenuates the cognitive deterioration in AD model mice. Moreover, rhLf treatment was efficient in protect brain from oxidative stress, as demonstrated by the marked decrease of ROS production due to increased levels of superoxide dismutase. At last, rhLf treatment significantly decreased both TNFα and IL-6 levels in the brain [138], confirming also in this pathology the Lf anti-inflammatory activity.

A recent clinical trial on the efficacy of oral cbLf treatment (250 mg/day for three months) against AD was also carried out. The cbLf treatment showed to be significantly efficient in decreasing serum IL-6 and increasing serum IL-10 [119]. Intriguingly, the results observed could indicate improved neuronal survival in cbLf treated AD patients compared to the control group. This evidence could be related to the iron-dependent antioxidant and to the iron-independent anti-inflammatory effects of Lf. Overall, these studies offer promising future approches for AD potential therapy.

### 3.4. Lactoferrin Against Inflammation in Type 2 Diabetes

Type 2 diabetes mellitus is a long-term metabolic disorder characterized by high blood sugar levels due to insulin resistance, which may be associated to its relatively reduced production. Insulin, a peptide hormone synthetized by beta cells of the pancreatic islets, promotes the absorption of carbohydrates, especially glucose from the blood into liver. The reduced sensitivity of body tissues to insulin is supposed to involve the insulin receptor and relative substrates. Moreover, inflammatory mediators, including some cytokines as TNF-α, IL-1 and IL-6, seem to be potential co-effectors of this pathology. Indeed, exposure of cells to TNF-α and IL-1 induces the inhibitory phosphorylation of serine residues of insulin receptor substrate (IRS-1) [139,140,141]. This phosphorylation reduces the ability of IRS-1 to associate with the insulin receptor thus inhibiting the insulin action [139,141,142]. IL-1 can also cause beta cells’ destruction in pancreatic islets by activating pathways involving nuclear factor-κB (NF-κB) and mitogen-activated protein kinase [143]. IL-6 impairs insulin activity in the three major insulin-responsive tissues: in the liver inhibits the insulin action on glycogen synthesis, in adipose tissue inhibits insulin activity on glucose transport and lipogenesis and in skeletal muscle stimulates insulin action on glucose transport [144]. Moreover, the daily macronutrients acquired, as glucose, contribute to inflammatory processes. Indeed, large quantities of glucose can cause an increase in superoxide generation [145] and an activation of intracellular NF-κB [146].

Numerous anti-inflammatory drugs have been evaluated for diabetes’ treatment in order to counteract the insulin resistance induced by inflammation. Among these, some clinical trials were conducted using anakinra (Kineret; Amgen), a recombinant human IL-1 receptor antagonist, able to either improve glycemia and beta cells’ secretory function as well as to down-regulate systemic inflammation markers [147,148]. Two studies conducted with canakinumab, another IL-1 antagonist, have shown increased insulin secretion levels [149,150]. In other studies, Salsalate (salicylate), a salicylic acid derived pro-drug, was demonstrated to be efficient in improving type 2 diabetes by inhibiting NF-κB pathway [151,152].

Recently, in a randomized clinical study, 60 children diagnosed with type 2 diabetes were randomly recruited and treated with 250 mg/day of camel Lf for 3 months. To investigate the potential therapeutic benefits on glycemia, the analysis of cytokines production as well as the evaluation of both inflammatory status and immunomodulatory effects were carried out [153].

In the current study, Lf treatment decreased diabetes-associated inflammation by inhibiting the Tool Like Receptor (TLR)-4–NF-κB axis with correlated reduction of serum pro-inflammatory cytokines, such as IL-1β, IL-6 and TNFα. Indeed, TLR-4 signaling activates NF-κB, that translocates into the nucleus, where activates the set of mentioned pro-inflammatory genes. As a consequence of this anti-inflammatory activity, a significant increase of insulin synthesis with a significant decrease of glycemia were observed [153]. These encouraging results indicate that the Lf supplementation may play an important role in restoring both the immunity balance and glycemic status in patients with type 2 diabetes.

## 4. Lactoferrin Against Septic Inflammation

The human body accommodates a large number of microorganisms that colonize and live in symbiosis with the host [154,155]. In particular, skin, oral cavity, urogenital tract, upper respiratory tract and intestine are strictly in contact with the environment and are colonized by microorganisms [156]. Commensal bacteria exert several defense functions, including the control of pathogenic microorganisms’ colonization by competing with common resources and by stimulating basal immune response thus inducing the production of the antimicrobial peptides by epithelial cells [156,157,158].

The relationship between humans and microbes is multifaceted: even if the vast majority of microorganisms are innocuous and beneficial to the host, some microorganisms can shift towards a pathogenic phenotype, defined as pathobiont [156,159], able to adhere to the host, invade underlying tissues and multiply thus evading host immune defenses [160]. The host responds to pathogens or pathogen-associated molecular patterns (PAMPs) producing inflammatory mediators, which, in turn, recruit mature neutrophils releasing granules-contained Lf in the sites of infection [25,161].

In addition to the natural defense systems, the efforts to counteract microbial infections are becoming vain over time due to the emergence of the antibiotic resistance as well as to different lifestyles that microorganisms assume duringthe infection (free, adherent, intracellular and in biofilm).Therefore, it is imperative to find new drugs or natural substances able to inhibit microbial replication or the biofilm formation or the microbial adhesiveness or invasiveness together with a significant anti-inflammatory activity. Lf, considered to be a part of the natural immune system, is localized in strategic positions as the mucosal surfaces [162], where, it exerts both an anti-microbial activity as well as an anti-inflammatory one, as demonstrated in several in vitro and in vivo studies [3,25,38,50,163]. In particular, in the context of septic inflammation, bLf has been found to be relevantly efficient in reducing the occurrence of neonatal late-onset sepsis in several clinical studies [164,165,166], recently reviewed by Telang [167].

### 4.1. Lactoferrin Against Inflammation Related to Chlamydia trachomatis Infection

The vagina of healthy childbearing age women is a complex ecosystem composed by several microbial species that establish a mutualistic relationship with the host.

These microorganisms compose the vaginal microbiota (VM) and co-participate to preserve the healthy status of the vagina as well as the maintenance of acidic pH (pH < 4.5) [168,169,170].

Vaginitis is an inflammatory condition that causes vaginal discharges, itching, irritation, burning and odor. Bacterial vaginosis, trichomoniasis and vulvovaginal candidiasis are the most common infectious causes of vaginitis [171,172,173].

Bacterial vaginosis (BV) is a pathologic condition characterized by dysbiosis of VM where a marked drop or disappearance of lactobacilli, the main components of commensal flora in the healthy vagina, is observed. In addition, an overgrowth of anaerobes, responsible for the activation of pro-inflammatory processes, including the release of pro-inflammatory mediators, such as IL-1β, IL-6, and IL-8 has been found [170,173,174,175,176,177].

BV represents a risk factor for tubal factor infertility, pelvic inflammatory disease, obstetrical complications and high susceptibility to sexually transmitted pathogens as *Chlamydia trachomatis*, *Neisseria gonorrhoeae* and *Trichomonas vaginalis* [178,179]. In particular, BV causes endometritis, cervical inflammation, infiltration of neutrophils and localized erythema. Furthermore, women affected by BV, as well as those with sexually transmitted bacterial infections (STBIs), in vaginal fluid (VF) present higher concentrations of IL-1β, IL-6 and IL-8, as well of Lf, thought to be produced in the cervical mucus by both cervical gland cells and neutrophils, compared with the healthy women [174,175,177,180].

The increase of Lf concentrations and its multi-functions represent an innate immune defence in the female reproductive tract [177]. The potential influence of bLf on VM and STBIs has been lately explored. Vaginal bLf administration to women affected by BV was able to change VM composition, reducing BV-related Gardnerella, Lachnospira and Prevotella genera and increasing *Lactobacillus* spp. [177].

Moreover, a positive effect of Lf in counteracting the STBIs due to *C. trachomatis*, *N. gonorrhoeae*, *Trichomonas* spp. or *Candida* spp. infection has been proven [50,177,181,182,183,184,185,186].

Worldwide, STBIs are commonly due to *C. trachomatis*, an obligate intracellular pathogen causing acute and chronic infections associated to inflammation. Like all intracellular pathogens, *C. trachomatis* requires essential intracellular nutrients, comprising iron, for its replication in host cells. In fact, iron-chelating compounds hinder the *C. trachomatis* replicative cycle [187]. In this respect, bLf has been used to counteract *C. trachomatis* infection [184]. Indeed, in in vitro study bLf interferes with *C. trachomatis* entry into human epithelial cell line when cell monolayers were incubated with bLf at the moment of the infection [184]. The inhibition of *C. trachomatis* infectivity, exerted by bLf, is probably due to its bind with host cells surface components, as glycosaminoglycans and heparan sulfate [54,188], which can be potential receptors for *C. trachomatis* adhesion [189]. In addition to the inhibition of bacterial entry into the cells, Lf exerts a potent anti-inflammatory activity down-regulating IL-6 and IL-8 synthesis by infected cells. In these experiments, bLf treatment was carried out 3 h after the infection in order to avoid any possible interference with *C. trachomatis* adhesive and invasive processes, thus excluding the possibility that results on bLf anti-inflammatory activity could have been due to different numbers of *C. trachomatis* inclusion forming units (IFUs) [184]. In another study, the anti-Chlamydial and anti-inflammatory activities exerted by bLf, alone or in combination with *Lactobacillus brevis* or *Lactobacillus crispatus*, were confirmed [185]. Of note, the combination of bLf with *Lactobacillus brevis* showed the highest anti-inflammatory effect, as demonstrated by the marked decrease of both IL-8 and IL-6 levels [185]. This evidence has demonstrated, once more, the marked bLf efficiency in down-regulating pro-inflammatory cytokines. These data were supported by results obtained in an in vivo pilot study on 7 pregnant women presenting *C. trachomatis* positive cervical specimens. The women were intravaginal treated with 100 mg of bLf every 8 h for 30 days. After bLf treatment, six out of seven pregnant women showed a significant decrease in cervico-vaginal IL-6 levels and, notably, resulted negative for *C. trachomatis* [184]. Even if further clinical studies are required, bLf can be an alternative candidate to treat *C. trachomatis* infection and inflammation.

### 4.2. Lactoferrin Against Inflammation Related to Cystic Fibrosis Lung Infection

Cystic fibrosis (CF) is a multi-system genetic disorder affecting several organs and reducing expectancy and quality of life. The most relevant damages are observed in the airways, that are characteristically susceptible to infections, principally, but not only, due to *Pseudomonas aeruginosa* [190,191]. During CF-induced airway infection progress, *P. aeruginosa* gradually shifts from the virulent form typical of early stage of infection towards the host-adapted form distinctive of the chronic phase, characterized by biofilm lifestyle, and antibiotic resistance [192].

Moreover, a massive airways inflammatory response is usually observed in CF subjects, which is already active before bacterial colonization, as evidenced by high levels of IL-8 and neutrophils’ accumulation in bronchoalveolar lavage (BAL) [193]. In general, the inflammatory status is activated by an over-expression of NF-κB and activator protein (AP)-1, leading to an up-expression of pro-inflammatory cytokines [194,195,196]. As a matter of fact, the massive recruitment of leukocytes into the lung airways gives rise to a self-enhancing loop, where the infiltrated neutrophils undergo necrosis and release proteases and chemoattractant molecules, thus leading to tissue damage and to the further recruitment of leukocytes.

Therefore, therapeutic strategies need to be addressed not only to counteract lung infection but also inflammatory processes and immunity disorders in CF patients.

In this respect, bLf has been used to decrease *P. aeruginosa* load and inflammation in in vitro and in vivo studies. In a paper by Frioni et al. [197], the addition of bLf to a primary human CF airway epithelium did not influence *P. aeruginosa* LESB58 adhesion, but significantly decreased intracellular bacterial number. In addition, bLf was able to reduce inflammatory response by infected bronchial epithelium by down-regulating the levels of IL-1β, IL-6 and IL-8 [197].

Interestingly, the bLf anti-inflammatory activity on CF infected bronchial (IB3-1) cells was also reported in the paper by Valenti et al [198], where cells were infected by *Burkordelia cenocepacea*, an opportunistic facultative pathogen involved in chronic lung infections and cepacia syndrome, frequently infecting CF patients. In particular, bLf was significantly efficient in decreasing the pro-inflammatory cytokines compared to untreated infected cells. This effect was correlated to the nuclear localization of bLf in infected IB3-1 cells, thus supporting its potential role in directly modulating gene expression [198].

These in vitro data on *P. aeruginosa* and *B. cenocepacea* have been confirmed in a recent in vivo study, where mice affected by acute and chronic *P. aeruginosa* lung infection were treated with aerosolized bLf [199]. In the acute infection model, mice underwent a sole intra-tracheal treatment with 100 or 200 µg aerosolized bLf, or saline solution as control. In the chronic infection model, mice underwent daily intra-tracheal treatment, for a total of seven treatments over six days, with 100 or 200 µg of freshly prepared bLf solutions, or saline as control. As a result, aerosolized bLf treatments reduced bacterial load in both acute and chronic infection models compared to the control. Notably, in chronic infection, the 18% of bLf-treated mice showed no *P. aeruginosa* in both BALs and lung homogenates, indicating a complete clearance of the infection [199]. Regarding immune response induced by infection, mice treated with either 100 µg or 200 µg bLf treatments showed a significant decrease of total leukocytes and neutrophils counts in BALs for both acute and chronic infection models, compared to saline treated ones. Moreover, both bLf dose treatments were efficient in differently decreasing the expressions of several pro-inflammatory cytokines, such as IL-1α, IL-1β, IL-6, KC (analogous to human IL-8), macrophage inflammatory protein (MIP-1α), granulocyte-colony stimulating factor (G-CSF), IL-12 (p40), and IL-12 (p70), depending from acute or chronic infection. Interestingly, the bLf-mediated decrease of KC, G-CSF and MIP-1α, molecules involved in neutrophil development and migration [197,200,201,202], is in agreement with the reduction in neutrophil recruitment, as demonstrated by the total and differential immune cell counts in BALs [199].

Overall, the aerosol administration of bLf in *P. aeruginosa* lung infected mice exerted several protective functions against both infection and inflammation by decreasing bacterial load, neutrophil recruitment and some pro-inflammatory cytokines/chemokines, thus globally improving mice health conditions.

## 5. Lactoferrin against Aseptic and Septic Inflammatory Bowel Disease

The inflammatory bowel diseases (IBD) include principally Crohn’s disease (CD) and ulcerative colitis (UC).

These pathologies are characterized by a loss of barrier integrity and inflammatory processes [203]. Intestinal barrier integrity is a key feature in the health of humans, especially for newborns, because the neonatal immature gastrointestinal system and immune system are still developing [204]. In addition, barrier integrity is crucial to prevent the entry of noxious luminal antigens from the intestinal lumen into mucosa and blood circulation [205]. Therefore, the loss of intestinal barrier integrity increases mucosal permeability and host susceptibility to pathogens, thus damaging the immune homeostasis as well as inducing inflammatory response [206]. In intestinal epithelial cells the tight junctions (TJs) play a pivotal role in barrier’s function. In particular, claudins, occludin, and zonula occludens, shaping TJs, maintain the epithelial barrier [207,208].

If the claudins, occludin, and zonula occludens synthesis decreases, the integrity of the barrier is disturbed and food allergy, asthma, as well as inflammatory processes can be triggered [205,209,210]. Defective barrier integrity and destructive inflammation, peculiar of CD, lead to the abnormal recruitment of immune cells, comprising neutrophils [206,211].

The direct evaluation of barrier integrity is generally diagnosed by endoscopy, which, however, can worsen the mucosal damage. On the contrary, the detection of fecal biomarkers is promising as a convenient, low cost, non-invasive diagnostic method, useful to monitor disease and subsequent relapse. In this respect, the detection of fecal Lf (fLf), resistant to degradation by proteolytic enzymes [212], present at high concentrations in the stomach and the small intestine, is a very actual and sensitive marker of IBD. Indeed, fecal levels of Lf rise quickly according to the influx of neutrophils during inflammation [205]. The fLf values range between 60 mg/g and 240 mg/g for active CD and 324 mg/g for active UC [213,214].

Other parameters, characterizing patients affected by CD and UC, are high levels of NF-κB, TNF-α, MCP-1 and IL-6 [215] related to a significant increase of M1 pro-inflammatory phenotypic macrophages [216]. Recently, bLf has emerged as an attractive molecule able to induce the shift from inflammatory to tolerogenic macrophage’s phenotype (Figure 1) [20]. In this respect, bLf appears to be a protective natural compound due to its anti-inflammatory activity able both to counteract the production of pro-inflammatory cytokines by epithelial cells and to drive the macrophagic shift toward the tolerogenic one [20,197].

The protective function of bLf in aseptic IBD has been reported in some in vitro and in vivo approaches. In a recent in vitro study, Zhao et al. [203] have demonstrated that bLf triggers the up-regulation of claudin-1, occludin, and zonula occludens-1 expression, which, in turn, strengthen the barrier function of intestinal cells. Interestingly, the decrease of TNF-α mRNA levels, in a bLf dose-dependent manner (50 µg/mL and 100 µg/mL), and the consequent inhibition of NF-κB signal pathway were also observed [203]. Concerning in vivo studies, Togawa et al. [217] demonstrated that in dextran sulfate sodium-induced colitis in rats, mimicking human IBD, the oral administration of Lf (200 mg/kg for 7 days) decreased inflammation and myeloperoxidase activity used as a biochemical marker of neutrophil infiltration.

On the other hand, only one in vitro study has been published on both the anti-microbial and anti-inflammatory efficacy of bLf in a model of septic IBD (197).

In this regard, we have to recall that the infections associated to IBD exacerbate immune stimulation, epithelial dysfunction and mucosal permeability. These disorders appear to be mainly related to the commensal-to-pathogenic shift of some bacterial species, especially *Escherichia coli* which enhances their mucosal adhesion, invasion, intracellular persistence and replication within macrophages [218,219]. Adherent invasive *E. coli* (AIEC) worsens the pathological destructive inflammation in CD patients [220,221,222,223]. Among AIEC strains, *E. coli* LF82 is considered as an important pathogen prototype infecting CD patients. In differentiated Caco-2 cells, LF82 is able to adhere through type 1 pili and carcinoembryonic antigen–related cell adhesion molecule (CEACAM-6) and to entry inside the host cells where it is able to replicate, survive [224,225] and induce inflammation [197]. In the above-mentioned in vitro study, the addition of bLf induces a decrease of invasion ability and bacterial survival together with a reduction of pro-inflammatory cytokines’ expression [197]. All these data strongly encourage the bLf use in the treatment of IBD.

## 6. Conflicting Data on Lactoferrin Anti-Inflammatory Activity in In Vitro versus In Vivo Models

Taken together all the data reported, Lf appears as a potent anti-inflammatory glycoprotein in in vivo studies. However, literature in vitro data report conflicting and contradictory results on the Lf role in inflammatory processes, ranging from pro- to anti-inflammatory activity. For instance, despite the numerous, above described, in vitro and in vivo studies on the Lf inhibitory effects on IL-6 production, some in vitro studies report a Lf-mediated up-regulation for this cytokine. In particular, Lf is described to induce IL-6 production in murine peritoneal macrophages through both a TLR-4 dependent and independent pathway [226,227]. Interestingly, bLf-treated monocyte-derived dendritic cells (MD-DCs) produce IL-6 [228]. On the contrary, bLf exposure to already differentiated MD-DCs totally fails to activate IL-6 production [228]. In this regard, Lf functions are influenced by in vitro cell differentiation status. Furthermore, the choice of different in vitro cell models (from phagocytic to epithelial cell lines), cell culture media and their relative iron and serum Tf content, as well as the stimulatory and infecting agents (from PAMPs to pathogenic bacteria), has delayed the actual and current recognition of the anti-inflammatory function of Lf. For instance, macrophages are highly responsive to both bacteria and PAMPs stimulation in the pathological production of IL-6 [19,20], whilst epithelial cell models are more responsive to intracellular pathogens than to PAMPs [184,197,198,199,229,230,231,232]. Moreover, the cell metabolic status greatly influences Lf functions. Two important factors must be taken into account: the cell receptor interactome, leading to the activation/inhibition of intracellular pathways, and Lf translocation into the nucleus. Moreover, the metabolic status is also influenced by cell aging, which activates a sort of auto-inflammatory process, resulting in inaccurate and non-reproducible results, very far from in vivo networks [20]. It is important to be aware that, when investigating Lf activities, several parameters influence the results, including its physico-chemical properties [36].

In fact, the use of different commercial or self-produced Lf preparations, showing unlike rates of purity, integrity, iron binding ability and iron or metal saturation rate, can be considered a further issue explaining the different contradictory results [36]. In particular, even if Suzuki et al. [58] have demonstrated the importance of the sole N-lobe, with respect to C-lobe, for Lf binding, internalization, and targeting to the nucleus, the integrity of cbLf preparation is essential to exert its anti-inflammatory activity.

Another pivotal parameter to be checked in cbLf preparations is the different degree of LPS contamination. Indeed, Lf-bound LPS has been found to interfere with inflammatory pathways in in vitro experiments [226]. Consequently, in order to define the actual pro-inflammatory activity of Lf, the pro-inflammatory cytokine concentration should be quantitatively detected thus distinguishing the physiological from the pathological inflammation. In this respect, the actual cytokines’ quantization by immunological assay, rather than the qualitative analysis through transcript or western blot detections, as well as the analysis of LPS contamination rate are strictly recommended.

From all the above, we can conclude that a meticulous quality analysis of commercial or self-produced Lf preparations as well as the choose of the most appropriate in vitro cell model and the type of inflammatory stimuli are of the utmost importance when studying cell responses to the immunomodulatory and anti-inflammatory functions of Lf.

## 7. Conclusions

In recent years, the interest in the use of natural substances endowed with anti-inflammatory activity is increasing. Indeed, anti-inflammatory drugs present several side effects especially in prolonged therapies. Among natural compounds, Lf, without side effects, has been shown to play a key role in counteracting inflammatory homeostasis disorders associated to both aseptic and septic pathological conditions. Summarizing, Lf is the sole glycoprotein able to contemporarily act against microbial multiplication, biofilm formation, iron disorders and oxidative stress, viral and parasitic infection as well as inflammation. Overall, the capacity of Lf to act as an anti-inflammatory glycoprotein is based on the reduction of pro-inflammatory cytokines involved in the complex orchestration of iron homeostasis mediated by the expression of several proteins as TfR1, Ftn, Fpn and Heph/Cp in inflamed epithelial and macrophagic cells, respectively. Host cells, depending on stimulating agents, express to different extents these genes leading to different outcomes depending on epithelial cell type and macrophage phenotype.

**Figure 2:**
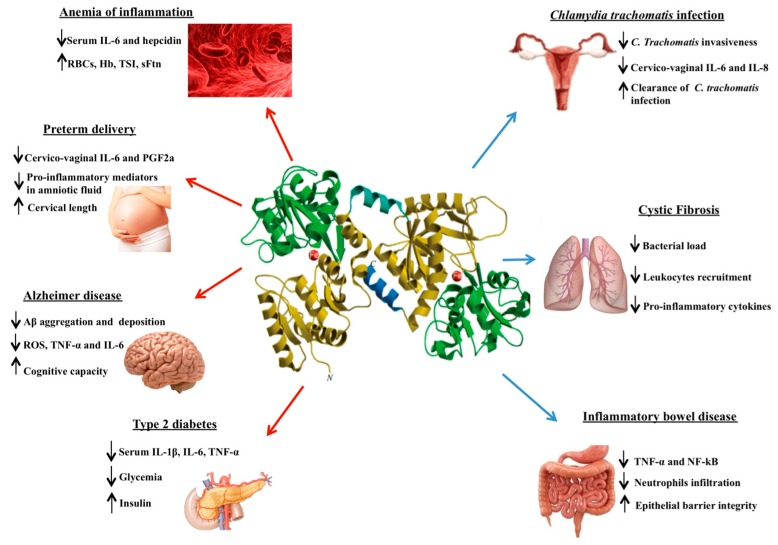
Schematic representation of the lactoferrin anti-inflammatory effects in different aseptic (red arrows) or septic (blue arrows) inflammatory pathologies. Lower and upper arrows indicate parameters decreased or increased, respectively.

Of note, Lf in physiological conditions does not exert any effect on epithelial and macrophagic cells while in pathological ones possesses “the ability to sense the immune activation status of an organism and act accordingly” [25]. Therefore, Lf can be an important tool in treating different pathologies as anemia of inflammation, preterm delivery, Alzheimer’s disease, type 2 diabetes and in counteracting intracellular iron overload and oxidative stress in infection processes associated with sexually transmitted diseases, as well as cystic fibrosis and inflammatory bowel disease, as summarized in Figure 2.
